# The Role of Leukocytes in Diabetic Cardiomyopathy

**DOI:** 10.3389/fphys.2018.01547

**Published:** 2018-11-01

**Authors:** Anamika Bajpai, Douglas G. Tilley

**Affiliations:** Center for Translational Medicine, Lewis Katz School of Medicine, Temple University, Philadelphia, PA, United States

**Keywords:** diabetes, heart failure, leukocyte, lymphocyte, inflammation

## Abstract

Diabetes is predominant risk factor for cardiovascular diseases such as myocardial infarction and heart failure. Recently, leukocytes, particularly neutrophils, macrophages, and lymphocytes, have become targets of investigation for their potential role in a number of chronic inflammatory diseases such as diabetes and heart failure. While leukocytes contribute significantly to the progression of diabetes and heart failure individually, understanding their participation in the pathogenesis of diabetic heart failure is much less understood. The present review summarizes the role of leukocytes in the complex interplay between diabetes and heart failure, which is critical to the discovery of new targeted therapies for diabetic cardiomyopathy.

## Introduction

Diabetes is a metabolic syndrome that manifests a low grade of systemic inflammation, leads to an increase in all-cause mortality and contributes to the development of number of cardiovascular complications (Duncan et al., 2003). Cardiovascular diseases remain the leading cause of deaths in the United States and in many countries globally, including coronary heart disease, stroke, high blood pressure, and arterial diseases (Benjamin et al., 2018). Notably, death rates among adults with both heart disease and diabetes mellitus are 2–4 times higher than those with heart disease alone, and the mortality rate of patients with heart disease >65 years of age is ∼68% in conjunction with diabetes (Benjamin et al., 2018). Clearly diabetes very negatively impacts the progression and outcome of heart disease, thus understanding the interplay between the two is an important endeavor for advancing treatment strategies of patients with diabetic cardiomyopathy (DCM).

The mechanisms contributing to diabetic cardiac dysfunction are complex and involve a number of molecular phenotypes including insulin resistance, oxidative/nitrative stress (Vita and Keaney, 2002; Creager et al., 2003; Widlansky et al., 2003), activation of mitogen-activated protein kinase (MAPK) (Malek et al., 1999; Vita, 2002), pro-inflammatory, poly (adenosine diphosphate [ADP]-ribose) polymerase (PARP) (Calles-Escandon and Cipolla, 2001), transcription factors (Kim et al., 2006; Bakker et al., 2009), as well as changes in the composition of extracellular matrix (Heil and Schaper, 2004) and inactivation of pro-survival pathways (Silver and Vita, 2006), eventually leading to cell death (Korshunov et al., 2007), which have been reviewed elsewhere (Jia et al., 2018). At a cellular level, high glucose levels negatively impact the function of several cell populations such as cardiac progenitor cells (Salabei et al., 2016), cardiomyocytes, adipocytes (Wang et al., 2006), fibroblasts (Russo and Frangogiannis, 2016) and leukocytes (Burke et al., 2004). For instance, higher levels of glucose and free fatty acids stress pancreatic islets and insulin-sensitive tissue such as adipose tissue, which leads to local production of the cytokines interleukin-1β (IL-1β), tumor necrosis factor-alpha (TNF-α) and chemokines CC-chemokine ligand 2 (CCL2), CCL3 and CXC-chemokine ligand 8 (CXCL8). Exposure to glucose also results in increased levels of advanced glycation (glycosylation or glycoxidation) end products (AGEs) that can directly regulate endothelial cell permeability, monocyte migration, and ultimately promotes inflammatory gene expression, contributing to microvascular and macrovascular complications (Goldin et al., 2006). Glucose levels also correlate with mitochondrial transmembrane potential in peripheral blood leukocytes attained from human Type I diabetics (Matteucci et al., 2011), an increase of which results in elevated superoxide production that may directly contribute to cell damage (Brownlee, 2001).

Numerous studies have shown that leukocytes and their subsets (neutrophils, monocytes, and lymphocytes) are involved in both the initiation and progression of cardiovascular diseases (Madjid et al., 2004; Hansson, 2005; Sarndahl et al., 2007). Diabetic cardiac injury is characterized by increased leukocyte mobilization and secreted pro-inflammatory cytokines, adhesion molecules, oxidative stress (Yu et al., 2011; Hernandez-Mijares et al., 2013) and stimulation of nuclear factor kappa-light-chain-enhancer of activated B cells (NF-κB) (Lorenzo et al., 2011). Higher leukocyte counts are associated with predicting the risk of cardiovascular disease in diabetic patients (Hong et al., 2014), suggesting a key role of these cells in worsening diabetes-associated cardiovascular disease.

A number of review articles have summarized the role of leukocytes in either diabetes or cardiovascular disease (for instance please refer to: Donath and Shoelson, 2011; Frangogiannis, 2014); however, increasing rates of heart failure in diabetic patients warrants an examination of the literature regarding the role of leukocytes in diabetic cardiovascular disease. Therefore, this review focuses on the role and behavior of leukocytes in the pathogenesis of diabetic heart failure.

## Leukocytes, Inflammation, and Diabetes

Leukocytes are essential mediators of the immune system that fight against foreign elements and maintain tissue homeostasis (Fearon and Locksley, 1996). Leukocytes work in an organized fashion with an impressive range of action (Odegaard and Chawla, 2008). They are derived from hematopoietic stem cells (progenitor cells) in the bone marrow. These pluripotent stem cells produce two distinct lineages: lymphoid progenitor cells and myeloid progenitor cells. Lymphoid progenitors are the precursors of T- and B- lymphocytes (T- and B-cells) and myeloid progenitors are the precursors of neutrophils, basophils, eosinophils, monocytes, macrophages, erythrocytes, dendritic cells, and platelets (Kondo, 2010). Monocytes/macrophages, neutrophils, and lymphocytes in particular have been demonstrated to both regulate and be impacted by the pathogenesis of diabetes (Hong et al., 2014).

Chronic inflammatory diseases, including diabetes, are characterized by dysfunctional and uncontrolled leukocyte behavior (Graves and Kayal, 2008; Swirski and Nahrendorf, 2013). Leukocyte recruitment is triggered by inflammation and they can produce a plethora of cytokines, chemokines, and reactive oxygen/nitrogen species to act systemically during diabetes (Naguib et al., 2004), and at local sites during myocardial infarction- or atherosclerosis-induced cardiac injury (Hansson and Libby, 2006; Eming et al., 2007), thereby contributing to sustained inflammation. Early inflammatory events in diabetes triggers the release of pro-inflammatory cytokines including TNF-α, IL-1β, and IL-6 (Medzhitov and Janeway, 2000), which gradually increase as the disease progresses (Pickup et al., 1997). Several studies have demonstrated that initial elevated levels circulating IL-6, plasminogen activator inhibitor-1 (PAI-1), C-reactive protein (CRP) and fibrinogen, are associated with the manifestation of diabetes (Pradhan et al., 2001; Festa et al., 2002; Meigs et al., 2004). Pro-inflammatory cytokines downregulate the major anabolic cascades involved in insulin signaling and impair glucose homeostasis (Hotamisligil et al., 1995; Lumeng et al., 2007b). In response to pro-inflammatory mediators, the endothelial lining of the microvasculature will increase expression of intracellular adhesion molecule 1 (ICAM-1) and/or vascular cell adhesion molecule (VCAM-1) that interact with leukocyte-expressed integrins to capture them and allow their migration to the injured area (Chan et al., 2001; Henderson et al., 2001). These inflammatory cascades are tightly regulated by nuclear transcription factors including NF-κB, a master molecule of inflammation and tissue hemostasis (Lawrence, 2009). NF-κB activation leads to or boosts the expression of cytokines, chemokines and adhesion molecules and more prominent leukocyte recruitment. Thus, the inflammatory cascades - from leukocyte activation to NF-κB stimulation – work in a positive feedback loop fashion (Monaco et al., 2004; Lawrence, 2009).

Many leukocyte subsets are involved in diabetes-associated chronic inflammation, in particular neutrophils, macrophages, and T-cells. Neutrophils react to and secrete higher levels of cytokines and growth factors in diabetic patients relative to healthy controls, including IL-8, IL-1β, TNF-α, and IL-1ra, which contribute to further migration of neutrophils to inflammatory sites, phagocytic activity, release of lytic proteases, production of reactive oxygen species and apoptosis (Werner and Grose, 2003; Komesu et al., 2004; Baum and Arpey, 2005; Hatanaka et al., 2006). The excessive production of cytokines and exacerbation of neutrophil and macrophage activation may contribute to further tissue damage and increased susceptibility to invasive microorganisms (Tennenberg et al., 1999).

Macrophages are well-established phagocytic cells, which renders them effective at the clearance of apoptotic and necrotic cells (Gordon, 2003; Gordon and Martinez, 2010), but exist along a continuum of phenotypes that makes them difficult to definitively classify. As such, various classifications exist including classically activated macrophages (CAMφs) vs. alternatively activated macrophages (AAMφs) (Gordon and Martinez, 2010), and the more broad pro-inflammatory (M1) vs. pro-reparative (M2) macrophages (Nahrendorf et al., 2007; Mosser and Edwards, 2008; Bajpai et al., 2018). Under diabetic conditions, macrophages are recruited into adipose tissue (AT) and activated via local cytokine secretion (TNF-α, IL-12, and IL-6) (Vachharajani and Granger, 2009), contributing to the establishment of an inflammatory profile and insulin resistance within the tissue. A deficiency of MCP-1 (CCL2) or CCR2 (CCL2 receptor) in mice results in the impairment of pro-inflammatory macrophage recruitment to adipose tissue, thus impeding the induction of insulin resistance (Kanda et al., 2006; Yu et al., 2006) and suggesting an important role for pro-inflammatory macrophages in the initiation and development of diabetes. Further, free fatty acids can be recognized by Toll-like receptors (TLRs), leading to the activation of macrophages, which release more TNF-α (Shi et al., 2006; Davis et al., 2008). TNF-α, one of the cytokines most abundantly secreted by CAMφs, has the ability to reduce the expression of important genes in the glucose regulation process, such as the glucose transporter GLUT-4 (Lumeng et al., 2007a); in fact, TNF-α receptor KO mice are resistant to diabetes stimulation (Uysal et al., 1997), suggesting the endocrine function of adipose tissue (AT) directly impacts the development of insulin resistance via recruitment and activation of CAMφs. Secretion of cytokines by CAMφs further activates the JNK and NF-κB signaling pathways in various leukocytes, thereby promoting the further production of IL-1β, TNF-α, and MCP-1 and increasing the expression of iNOS, all of which contribute to insulin resistance in different tissues (Kaneto et al., 2005a,b; Andreasen et al., 2011). Myeloid-specific Iκκ-β (an activator of NF-κB)-deficient mice have shown decreased NF-κB activation and pro-inflammatory cytokine production (IL-1β, IL-6, TNF-α, and MCP-1), leading to inhibition of the development of insulin resistance (Arkan et al., 2005). Of note, it has been shown that IL-10 produced by AAMφs blocks the pathological effects of TNF-α in AT (Lumeng et al., 2007b; Prieur et al., 2011), suggesting that while CAMφs have insulin resistance-inducing effects, AAMφs have a protector role within AT. Indeed, A-ZIP transgenic mice (that are insulin-resistant and hyperlipidemic), which have a deficiency in MCP-1, displayed decreased hyperglycemia, hyperinsulinemia, and hepatomegaly; moreover, these mice had increased levels of AAMφs markers, such as Arg1 and Chi313 (Nio et al., 2012). Notably, AAMφ development is dependent on IL-4/IL-13 stimulation, which activates the transcription factor STAT-6, and STAT-6-deficient mice are more prone to obesity, oxidative stress in their AT and susceptibility to T2D development, which, in turn, is associated with the absence of AAMφs (Ricardo-Gonzalez et al., 2010).

Recent studies suggest adaptive immune cells, especially T lymphocytes, also play a pivotal role in diabetes. As with macrophages, CD4^+^ effector T cells can be divided into proinflammatory Th1, Th17, and anti-inflammatory Th2 and Foxp3^+^ regulatory T cell (Treg) subtypes based on their functionality and cytokine production (Raphael et al., 2015). Once activated, Th1 and Th2 cells show many significant signs of inflammation, such as cytokine release. For instance, Th1 cells produce interferon gamma- (IFN-γ), interleukin-2 (IL-2), and tumor necrosis factor beta (TNF-β), triggering cell-mediated immunity and phagocyte-dependent inflammation (Raphael et al., 2015). Th2 cells, in contrast, produce IL-4, IL-5, IL-6, IL-9, IL-10, and IL-13 to regulate antibody responses (Kahn et al., 2006). Studies have shown that Th1 and Th2 cells have key functional roles in regulating inflammatory processes, although they are activated later than macrophages during inflammation (Cintra et al., 2008; Martinez et al., 2008). Th17 cells, important pro-inflammatory CD4^+^ T cell subtypes that secrete IL-17 and IL-22, have also been associated with diabetes (Zuniga et al., 2010; Zhang et al., 2014). It was shown that macrophages from AT express the IL-22 receptor (IL-22R) and respond to Th17-released IL-22 to secret more IL-1β, thereby further promoting AT inflammation (Dalmas et al., 2014; Zhao R. et al., 2014). In all, leukocytes clearly contribute to the pathogenesis of diabetes, and herein we will discuss the impact of leukocyte regulation in diabetic cardiomyopathy.

## Leukocytes in Diabetic Cardiomyopathy

Heart failure associated with diabetes, or DCM, is a common hallmark of diabetes progression. As discussed above, diabetes is associated with chronic systemic inflammation, which leads to leukocyte activation and recruitment to various organs and further inflammatory tissue remodeling over time. In general, this results in organ fibrosis as resident fibroblasts become activated in response to pathophysiologic conditions, which for the heart leads to wall stiffening and decreased contractility (Russo and Frangogiannis, 2016). Reduced cardiac output ultimately stimulates further cardiac inflammation and fibrosis, leading to dilation and established heart failure. Leukocytes are known to modulate cardiac fibroblasts by virtue of secreted mediators of fibrosis, including transforming growth factor-β (TGF-β) (Bugger and Abel, 2014; Russo and Frangogiannis, 2016), however, whether DCM-induced fibrosis is preceded by leukocyte infiltration and activation has not been reported.

Several factors contribute to DCM and the potential leukocyte responsiveness during its progression, including chronic hyperglycemia, which leads to obesity, high cholesterol levels, as well as high blood pressure and coronary artery diseases. Recent evidence suggests cross-talk between inflammation and insulin signaling, highlighting a strong relationship between insulin-resistant states, inflammation, and heart failure (Kim et al., 2005). For example, altered microvascular endothelial ICAM-1 expression in diabetic rats has been shown to be restored with insulin treatment (Anjos-Valotta et al., 2006). There are also multiple molecular pathways involved in the induction of diabetic heart failure including oxidative/nitrative stress (Vita and Keaney, 2002; Creager et al., 2003; Widlansky et al., 2003), activation of mitogen-activated protein kinase (MAPK) (Malek et al., 1999; Vita, 2002), pro-inflammatory, poly (adenosine diphosphate [ADP]-ribose) polymerase (PARP) (Calles-Escandon and Cipolla, 2001) and transcription factors signaling pathways (Kim et al., 2006; Bakker et al., 2009), as well as changes in the composition of extracellular matrix (Heil and Schaper, 2004) and inactivation of pro-survival pathways (Silver and Vita, 2006).

**FIGURE 1:**
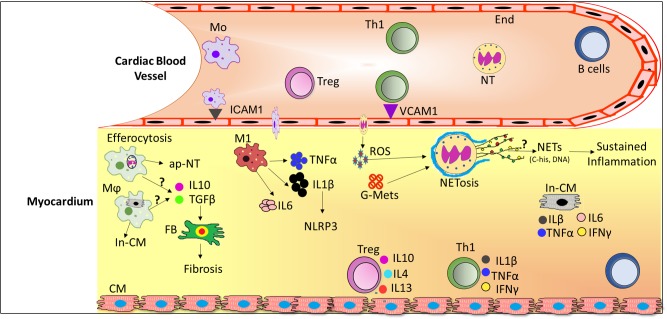
Schematic diagram depicting infiltration of leukocytes from the circulation and their role in the diabetic cardiomyopathy (DCM). In DCM, a number of local processes are activated by glucose metabolites, reactive oxygen species (ROS) and pro-inflammatory cytokines together with accumulation of neutrophils and macrophages into the lesion site. Upon infiltration, neutrophils release extracellular traps (NETs) which induce sustained inflammation. Activated macrophages phagocytose cellular debris and also release pro-inflammatory cytokines and growth factors which activates fibroblasts to induce fibrosis. Th1 cells secrete pro-inflammatory cytokines which further exacerbate the inflammation in DCM whereas Treg cells secrete anti-inflammatory cytokines, where the ratio of pro-/anti-inflammatory cytokines may predict the progression of DCM. Abbreviations: ap-NT, apoptotic neutrophils; B cells, B lymphocytes; CM, cardiomyocytes; End, endothelial cells; FB, Fibroblast; G-Mets, Glucose metabolites; In-CM, Injured cardiomyocytes; ICAM1, Intracellular adhesion molecule 1; IL6, Interleukin 6; IL1β, Interleukin 1 beta; IFNγ, Interferon gamma; M1, pro-inflammatory macrophages; Mo, monocytes; Mϕ, activated macrophages; NETs, Neutrophils extracellular traps; NT, neutrophils; ROS, reactive oxygen species; Th1, T helper cells 1; Treg, T regulatory cells; VCAM1, vascular cell adhesion molecule 1.

In the early phase of inflammation, proinflammatory cytokines including TNFα, IL-6 (Dinh et al., 2009) IL-1β (Masters et al., 2011), Interferon (IFN)-γ, TGF-β (Biernacka et al., 2015 are secreted by macrophages and/or lymphocytes and may cause or exacerbate cardiac injury. In addition, these locally produced cytokines have been found to possess autocrine and paracrine properties that can influence neighboring tissues to enhance vascular permeability (Salt et al., 2003), recruitment of invasive leukocytes (Hokama et al., 2000; Pettersson et al., 2011) and reactive oxygen species (ROS) production (Giacco and Brownlee, 2010; Mann, 2015; Low Wang et al., 2016). Altogether, disturbances in metabolic and inflammatory signaling pathways during diabetes progression are associated with alterations in leukocyte activation and enhanced cardiac inflammation (Figure 1). Therefore, in this section of review, we will discuss the role of leukocytes subsets in DCM.

### Neutrophils

Neutrophils often provide the first line of defense at sites of inflammation. These are considered short-lived effector cells, possessing limited capacity for biosynthetic activity and ROS generation, but have been shown to be crucially involved in cardiac repair by polarizing macrophages toward a reparative phenotype (Horckmans et al., 2017). In addition, they secrete a number of factors that regulate inflammation, including peroxidases, cytokines, microparticles (MPs), and neutrophil extracellular traps (NETs). The activity of myeloperoxidase (MPO), stored in azurophilic granules of neutrophils and released during inflammation (Anatoliotakis et al., 2013), has been shown to be increased in the plasma of patients with diabetes concomitant with coronary heart disease (Gorudko et al., 2012). Neutrophil gelatinase-associated lipocalin (NGAL) is one of the cytokines solely produced by neutrophils and its expression is increased following acute myocardial infarction and during chronic heart failure (Yndestad et al., 2009; Villacorta et al., 2015). NGAL modulates the enzymatic activity of matrix metalloproteinase-9 (MMP-9) and is an important mediator of plaque instability in atherosclerosis, suggesting that it might play a role in thrombo-inflammation (Sivalingam et al., 2017). MPs are small vesicles (0.1–1.0 μm) released from stimulated and/or apoptotic endothelial cells, platelets, and leukocytes (monocytes and neutrophils) (Boulanger et al., 2017). Neutrophil-derived MPs, which can be regulated by endothelium-derived MPs and depend on locally released nitric oxide (Muller, 2014), contain the functionally active anti-inflammatory protein annexin 1, which inhibits the interaction between leukocytes and endothelial cells *in vitro* and *in vivo* (Hayhoe et al., 2006; Sugimoto et al., 2016). The changes and roles of MPs in either diabetes, heart failure or DCM remains largely unknown.

A recently identified process involving NET formation, which involves the release of DNA and granule proteins of neutrophils that prime other immune cells to augment inflammation, may contribute to the development of DCM since studies have indicated that NET formation is enhanced in diabetic patients and ultimately contributes to impaired wound healing (Papayannopoulos, 2015; Wong et al., 2015). The release of NETs, termed NETosis, is a proposed cell death mechanism, which, if dysregulated, can contribute to pathogenesis (Fadini et al., 2016; Papayannopoulos, 2018). During NETosis, mitochondrial ROS, inflammatory cytokines and glucose metabolites may each participate in the activation of NF-κB to transcriptionally up-regulate peptidyl arginine deiminase 4 (PAD-4), which acts to promote histone processing, an important event in NET formation (Azroyan et al., 2015; Wong et al., 2015). Subsequently the digestion products and granule proteins contents are released into the extracellular space, providing an extremely strong pro-inflammatory stimulus (Wong et al., 2015; Silk et al., 2017). Future studies will be required to determine the specific impact of NETosis in diabetes progression, and more specifically in DCM.

### Macrophages

Macrophages have been implicated in the pathogenesis of diabetes, wherein they display impaired phagocytic activity (Tan et al., 1975; Khanna et al., 2010), reduced release of lysosomal enzymes (McManus et al., 2001), and reduced chemotactic activity (Khanna et al., 2010; Raj et al., 2018) in diabetic patients. These traits are significantly correlated with increased blood glucose levels (Jakelic et al., 1995) and reversed by decreasing blood glucose levels in both humans (Jakelic et al., 1995) and rats (Alba-Loureiro et al., 2006). Normally in injured tissue, macrophages engulf apoptotic cells and cellular debris to reduce inflammation, a phenomenon called efferocytosis (DeBerge et al., 2017). Several molecular processes contribute to this mechanism and in particular the metalloproteinase disintegrin and metalloproteinase domain-containing protein 9 (ADAM-9) was shown to be upregulated in macrophages under conditions of high glucose, secondary to decreased expression of miR-126, which increased MER proto-oncogene, tyrosine kinase (MerTK) cleavage to ultimately reduce efferocytosis (Suresh Babu et al., 2016). Importantly, human diabetic hearts displayed the same molecular signatures in terms of miR-126, ADAM9, and cleaved MerTK expression, suggesting this process may be involved in regulating human DCM progression. Thus, impaired efferocytosis would be expected to prolong cardiac inflammation as dead cardiomyocytes and debris would not be efficiently removed.

As discussed above, macrophages have been demonstrated to exist along a spectrum of phenotypes book-ended by either pro-inflammatory (M1) or pro-reparative (M2) descriptors, and certainly a regulated balance between the two subtypes is necessary for homeostasis of inflammation (Nahrendorf et al., 2007; Mosser and Edwards, 2008; Bajpai et al., 2018). During diabetes the balance favors the M1 phenotype, which acts to promote a low level of chronic tissue inflammation and insulin resistance (Rao et al., 2014). M1 macrophages have been shown to be upregulated in the myocardium prior to the onset of cardiac dysfunction (Nahrendorf et al., 2007) and early non-selective macrophage depletion with clodronate liposomes has been demonstrated to reduce cardiac inflammation (Schilling et al., 2012). Conversely, macrophages of the M2 phenotype are associated with reduced cardiac inflammation under conditions of experimental diabetes (Jadhav et al., 2013), however, further investigation is required to elucidate the impact of phenotype-specific depletion or activation of macrophages in the context of DCM. Notably, the M1 and M2 classification system is now thought to be oversimplified, with recognition of a spectrum of multiple macrophage phenotypes (Xue et al., 2014) that have been recently identified and which have unknown impact on DCM.

### T-Lymphocytes

Distinct T-lymphocytes subtypes, including T-helper subsets (Th) and T regulatory cells (Treg), regulate inflammation and insulin resistance. Increased frequency of Th1, Th17, and Th22 subsets were shown to contribute to coronary artery disease onset in diabetic patients after adjusting for age, sex, and duration of diabetes (Zhao R.X. et al., 2014). In another study, increased serum levels of Th1-associated cytokines (IL-12 and IFN-γ) with strong suppression of Th2-associated cytokines (IL-4, -5) were found to be correlated with diabetic coronary artery disease (Madhumitha et al., 2014). Several clinical studies have confirmed that Th1-associated cytokines are upregulated in the peripheral blood from pre-diabetic or T2DM (type 2 diabetes) patients (Zeng et al., 2012; McLaughlin et al., 2014), whereas the activation of Th2 cell-mediated immunity is delayed and impaired in diabetes (Wu et al., 2011). IL17- secreting Th17 cells are also increased in T2DM patients and may be associated with dysregulated lipid metabolism (Zuniga et al., 2010; Zhang et al., 2014; Garidou et al., 2015).

As their name suggests, Treg cells regulate inflammatory responses and tissue impairment (Sakaguchi et al., 2008; Nosbaum et al., 2016). In T2DM, Treg cells can suppress Th1, Th2, and Th17 responses by various pathways, such as the suppression of cytokine secretion, modulation of the microenvironment, and altering the expression of surface receptors to improve insulin resistance (Guzman-Flores et al., 2013; Bluestone et al., 2015). Foxp3^+^ Treg cells have been demonstrated effective in the control of autoimmune disease (Buckner, 2010), and in DCM patients, a significant reduction in peripheral TGF-β and IL-10 with decreased Foxp3 expression contributed to an imbalance in the Treg/Th17 ratio (Li et al., 2010, 2017; Tang et al., 2010). Given the decreased number of Treg cells (Jagannathan-Bogdan et al., 2011), as well as altered Treg/Th17 and Treg/Th1 ratios in patients with T2DM (Zeng et al., 2012), an appropriate balance between pro-inflammatory (Th17 or Th1) and regulatory (Treg) subsets of T cells may be required to maintain overall T cell homeostasis and prevent chronic inflammation. While it is evident that T cells play an important role in mediating cardiac injury (Bansal et al., 2017), and genetic depletion of T cells protects against cardiac fibrosis and decreased LV function (Laroumanie et al., 2014; Weirather et al., 2014; Nevers et al., 2015), further delineation of the role of each T-lymphocyte subset would be worthwhile exploring specifically in the context of diabetic heart failure.

### B-Lymphocytes

B-lymphocytes are antigen-presenting cells and autoantibody secretors. B-lymphocyte-deficient mice demonstrated less inflammation and exhibited improved glucose tolerance (Winer et al., 2011). Additionally, Nishimura et al. demonstrated that mice deficient of programmed cell death protein-1 (PD-1^-/-^, a key factor for B-cell differentiation) expressed elevated levels of circulating autoantibodies that bound specifically to cardiomyocytes and were associated with progression of dilated cardiomyopathy (Nishimura et al., 2001). In another study, B cells from diabetes mellitus patients had elevated pro-inflammatory IL-8 levels but failed to secrete the anti-inflammatory IL-10 under a variety of pro-inflammatory conditions (Jagannathan et al., 2011). In contrast, a recent study demonstrated that naturally occurring B-regulatory cells mediate protection against autoimmune destruction of pancreatic islets by selectively suppressing autoreactive T-cell responses (Kleffel et al., 2015). Given that B cells are the earliest cell type that infiltrate pancreatic islets in mice and directly regulate islet T cell infiltration, B cell-directed therapy could be effective to protect against diabetes, however, much more insight into their action under these conditions is required.

## Therapeutic Strategies

Since numerous signaling pathways activated during DCM ultimately contribute to fibrosis, preclinical studies have focused on mitigating this effect via targeting of various fibrogenic aspects. Several studies by the Tschöpe group showed that pre-clinical streptozotocin-induced DCM rodent models are associated with increased pro-inflammatory cytokine and adhesion molecule expression in the heart, as well as leukocyte accumulation and fibrosis, effects that were sensitive to treatment with a variety of treatments, including statin, interleukin converting enzyme inhibitor and monoclonal antibody-mediated inhibition of TNFα (Van Linthout et al., 2007; Westermann et al., 2007a,b). In addition, another group previously demonstrated that the antifibrotic agent tranilast, and its derivatives FT011 and FT23, act to oppose TGFβ-mediated fibrosis in a streptozotocin-induced transgenic (mRen-2)27 hypertensive rat model of DCM (Martin et al., 2005; Kelly et al., 2007; Tan et al., 2012; Zhang et al., 2012). These compounds acted to attenuate diastolic cardiac dysfunction, which was associated with decreased fibrosis and, notably, macrophage accumulation within the myocardium. Since therapeutic strategies for the treatment of cardiac fibrosis have been reviewed elsewhere (Russo and Frangogiannis, 2016), here we focus more specifically on clinical and preclinical evidence for potential therapies that could mitigate DCM via regulation of leukocytes themselves. As discussed above, both neutrophils and B-lymphocytes may offer potential therapeutic targets for the treatment of DCM, however, more preclinical studies will be required to assess this concept. As such, the remainder of the discussion will focus on reported responses to therapeutic strategies involving modulation of macrophage and T cell activities.

### Macrophages

Although inhibition of pro-fibrotic processes appears capable of decreasing the progression of DCM and cardiac leukocyte accumulation, reduced leukocyte accumulation within the diabetic heart has conversely been demonstrated to decrease cardiac fibrosis during experimental diabetes in rodents. For instance, treatment of either streptozotocin-induced mice, as a model for Type I diabetes, or Israeli sand rats, as a model for Type II diabetic cardiomyopathy, with the CXCR4 antagonist AMD3100 was able to decrease fibrosis, suggesting that inhibition of leukocyte recruitment to the heart during development of DCM is sufficient to decrease pro-fibrotic signaling (Chu et al., 2015). Additionally, a recent study reported that β2-adrenergic receptor (β2AR) stimulation of macrophages under conditions of high glucose inhibited pro-inflammatory NF-κB-dependent production of TNFα and that long-term treatment of Zucker diabetic fatty (ZDF) rats with the β2AR agonist salbutamol decreased monocyte activation, cardiac macrophage, collagen and fibronectin accumulation, as well as preserved cardiac function compared to non-salbutamol-treated ZDF rats (Noh et al., 2017). Notably, β2AR stimulation-mediated inhibition of macrophage activation *in vitro* and cardiomyopathy progression *in vivo* was context-dependent, occurring only under hyperglycemic but not normal glucose conditions, while our own studies have shown that β2AR agonism increases, while antagonism or deletion decreases, leukocyte responsiveness (Grisanti et al., 2016a,b). Thus, disease-specific environmental factors may play a key role in determining the effectiveness of potential therapeutics.

Additional studies support the involvement of macrophages in DCM, wherein clodronate-liposome-mediated depletion of macrophages was demonstrated to reduce the expression of macrophage and inflammatory markers in the heart and partially preserve cardiac function in a transgenic mouse model of cardiac lipotoxity (Schilling et al., 2012). Further, in streptozotocin-treated mice, pro-inflammatory cytokine expression, oxidative stress, fibrosis and cardiac dysfunction were associated with enhanced monocyte accumulation within the heart, all of which were reduced by treatment with bone morphogenetic protein 7 (BMP7), the supposition being that this promoted monocyte conversion into anti-inflammatory macrophages favoring survival signaling (Urbina and Singla, 2014). Similarly, fibroblast growth factor-9 administration to infarcted db/db diabetic mice was shown to enhance M2 macrophage polarization, which was associated with decreased inflammatory cytokine expression, reduced cardiac remodeling and improved cardiac function (Singla et al., 2015). Further, activation of peroxisome proliferator–activated receptor gamma (PPARγ), a ligand-activated transcription factor that controls the expression of key genes involved in lipid and glucose metabolism and inflammation (Blaschke et al., 2006), has been shown to reduce human monocyte chemotaxis (Kintscher et al., 2000) and suppress macrophage pro-atherosclerotic osteopontin expression (Oyama et al., 2002), suggesting that clinically used glitazones may be able to reduce the infiltration or phenotypic conversion of pro-inflammatory macrophages.

A more recent study similarly reported alterations in streptozotocin-treated mouse hearts, including enhanced pro-inflammatory cytokine expression, fibrosis and decreased function that was associated with macrophage accumulation, but notably highlighted the negative impact of estrogen deficiency on these processes through the use of ovariectomized female mice (Jia et al., 2017). These changes were also associated with increased expression of pro-M1/anti-M2 macrophage miR155. However, exacerbation of DCM in the absence of estrogen was prevented via either clodronat liposome-mediated macrophage depletion or treatment with gold nanoparticle-conjugated antago-Mir155, which promoted M2 macrophage marker expression and improved cardiac structure and function. Finally, induction of heme oxygenase-1 (HO-1) was shown to enhance M2 macrophage polarization *in vitro* and in rodent models, including high fat diet-fed C57BL/6 mice and ZDF rats, which led to the amelioration of pro-inflammatory cytokine generation and cardiac dysfunction in the face of diabetic cardiomyopathy (Sierra-Filardi et al., 2010; Jadhav et al., 2013; Tu et al., 2014). Altogether, these studies suggest that a balance between M1 and M2 macrophage phenotypes within the heart may be an essential component of controlling DCM progression.

### T-Lymphocytes

Similar to targeting macrophages, studies have highlighted the potential therapeutic effectiveness of targeting T lymphocytes for preventing the development of DCM. For instance, streptozotocin-treated mice displayed enhanced cardiac T cell infiltration associated with increased fibrosis and decreased cardiac function, each of which were augmented by T cell-specific deletion of hypoxia inducible factor 1α (HIF-1α) (Lin et al., 2016). Further, genetic depletion of T cell trafficking protected cardiac fibrosis and LV function by reducing S1P1 and TGF-β1 expression (Laroumanie et al., 2014; Weirather et al., 2014; Nevers et al., 2015). Additionally, Rag1KO mice, which lack mature T lymphocytes, are protected against streptozotocin-induced cardiac fibrosis (Abdullah et al., 2016). The same group has also reported that T-cell-specific sphingosine 1-phosphate receptor 1 (S1P1)-mediated signaling is essential for the streptozotocin-induced fibrosis as the S1PR1 antagonist FTY720 was able to attenuate this response, as was T cell-specific deletion of S1PR1 (Abdullah et al., 2016; Abdullah and Jin, 2018). Notably, while depletion of T cell-specific expression of S1PR1 exerted protection against cardiac fibrosis in the diabetic model, non-streptozotocin-treated T cell-specific S1PR1 knockout mice exhibited enhanced cardiac fibrosis, suggesting that S1P1R-dependent T lymphocyte signaling differentially alters cardiac remodeling outcomes in a pathologically contextual manner.

## Future Perspectives and Unanswered Questions

Although scientists have explored new phenotypes and functions of leukocytes in the context of heart failure, their role in diabetic cardiomyopathy is still developing and there remain several important avenues of research for the future. First, although the role(s) of leukocytes in regulating DCM in different experimental rodent models may overlay, the predominant use of the streptozotocin-induced Type I diabetes rodent model to investigate the leukocytes in the development and progression of DCM potentially leads to limited applicability to the clinically relevant and highly prevalent type II diabetes-associated DCM (Holscher et al., 2016). Thus, further studies are required to understand the potential differences in leukocyte phenotypes and their underlying mechanisms for promoting DCM using rodent models that better mimic conditions observed during the development of type II diabetes mellitus. Second, B-lymphocytes clearly contribute to cardiac remodeling during the development of heart failure since systemic B-lymphocyte depletion has been shown to reduce T cell–, macrophage- and neutrophil-induced tissue damage by reducing the systemic amplification of the inflammatory response after myocardial infarction (Zouggari et al., 2013). However, the role of B-lymphocytes specifically in the progression of DCM is unknown, therefore additional studies within this context are needed. Third, there are known differences between males and females in the progression of DCM (Natarajan et al., 2003; Laverty et al., 2017). It is evident that females are protected from cardiovascular diseases due to multiples factors including estrogen receptor signaling (Pare et al., 2002), reduced ROS production, and higher antioxidants (Barp et al., 2002; Ide et al., 2002). As such, future work would be immensely beneficial in understanding potential sex-specific leukocyte behaviors during the development and progression of DCM.

## Author Contributions

AB and DT wrote the manuscript.

## Conflict of Interest Statement

The authors declare that the research was conducted in the absence of any commercial or financial relationships that could be construed as a potential conflict of interest.
